# Evisceration of umbilical content with extensive adhesion: A surgical approach

**DOI:** 10.1002/ccr3.3217

**Published:** 2020-08-09

**Authors:** Pravin Mishra, Vivek Kumar Yadav, Moinul Hasan

**Affiliations:** ^1^ Faculty of Veterinary Science Bangladesh Agricultural University Mymensingh Bangladesh; ^2^ Department of Surgery and Obstetrics Bangladesh Agricultural University Mymensingh, Mymensingh Bangladesh

**Keywords:** electrocauterization, evisceration, umbilical hernia

## Abstract

With timely recognition and surgical intervention along with administration of an antibiotic, antihistaminic, and anti‐inflammatory drugs, evisceration of umbilical content with extensive adhesion can be treated, and the outcome is also good.

## Graphical abstract

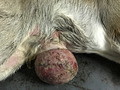

## 1 INTRODUCTION

Umbilical hernia is occasionally seen with different levels of complexity in animals. However, eviscerated umbilical hernia with extensive adhesion in a bovine calf is not much common. With timely recognition, surgical intervention, and administration of an antibiotic, antihistaminic, and anti‐inflammatory drugs, the outcome is generally good.

**FIGURE 1:**
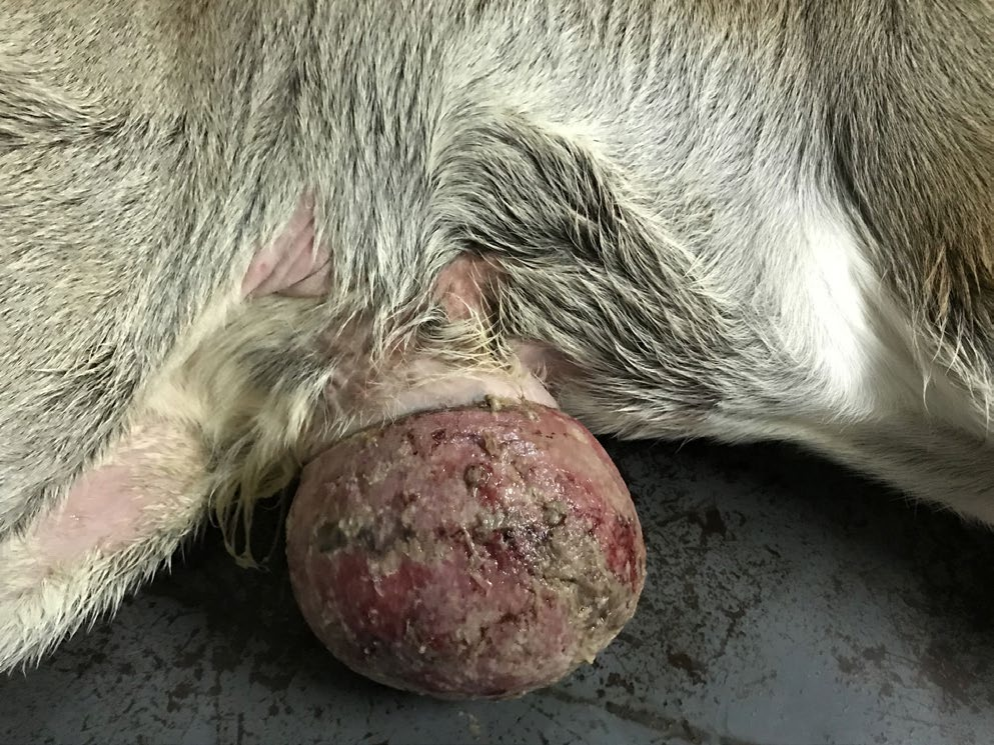
Evisceration of umbilical content with extensive adhesion in bovine calf

Twenty days old indigenous male bovine calf having body weight 40 kg presented with the complaint of swollen umbilical contents at Veterinary Teaching Hospital, Bangladesh Agricultural University, Mymensingh, Bangladesh. Physical examination confirms the case is an evisceration of umbilical content with extensive adhesion which is not much common in the bovine calf (Figure 1). All the other clinical parameters were found normal, which confirmed the calf was free from another infectious disease.

**FIGURE 2:**
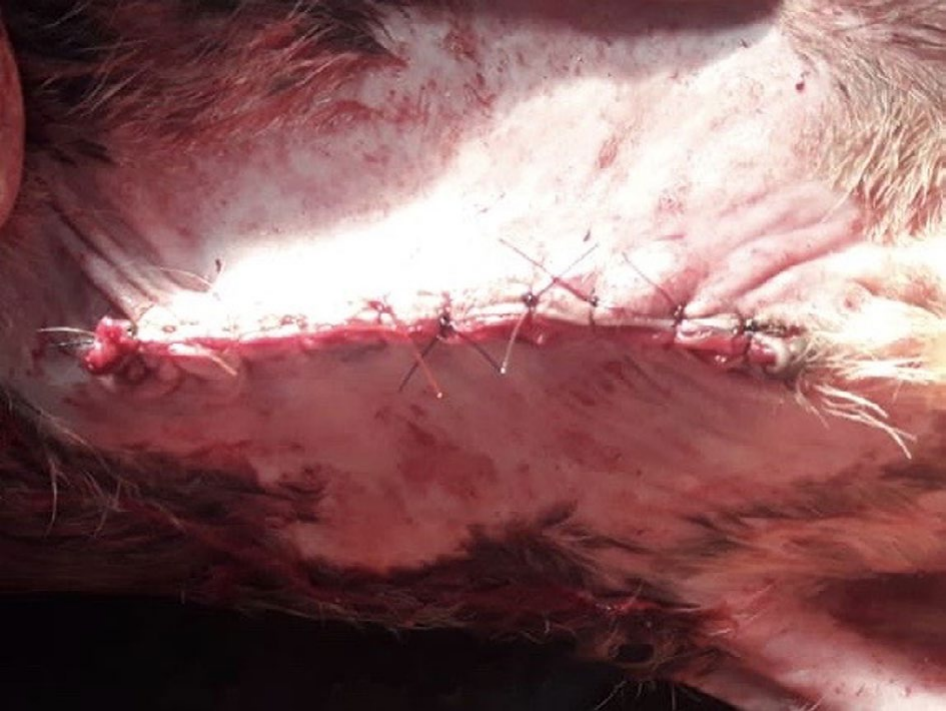
Simple interrupted suture on skin using nylon

Umbilical herniorrhaphy was done where electrocauterizer was used to incise the eviscerated tissue, which contains the part of the small intestine and also to minimize the bleedings. All the contents were put back in the abdominal cavity. Finally, the ring, muscle, and skin were sutured separately (layer by layer) with polyglactin 910 (No. 1‐0), catgut (No. 1‐0), and nylon (No. 1) (Figure 2), respectively. Along with the surgical process, administration of antibiotic, antihistamine, and anti‐inflammatory medication was done, and proper hygienic management was suggested. Now the calf is healthy and doing well.

The evisceration of the intestine may occur spontaneously after gradual worsening of the hernia and thinning of the wall, or it may be abruptly caused by trauma or sudden straining.^1^ With the timely surgical intervention and proper hygienic management of umbilical hernia, the outcome is generally good, otherwise recurrence or secondary infections may be seen as the chance of bacterial infection is high in male calves because of their anatomical structures.^2^, ^3^

## CONFLICT OF INTEREST

The authors declare that there is no conflict of interest regarding the publication of this manuscript.

## AUTHOR CONTRIBUTIONS

PM and MH: diagnosed the case and performed the surgery. PM and VKY: wrote the manuscript. All authors: revised the manuscript critically and approved the final version to be published.

## ETHICAL APPROVAL

Ethics approval was not required for this study.
